# Markers of potassium homeostasis in salt losing tubulopathies- associations with hyperaldosteronism and hypomagnesemia

**DOI:** 10.1186/s12882-020-01905-7

**Published:** 2020-07-06

**Authors:** Michael Eder, Elisabeth Darmann, Maria C. Haller, Marija Bojic, Markus Peck-Radosavljevic, Rainer Huditz, Gregor Bond, Andreas Vychytil, Roman Reindl-Schwaighofer, Željko Kikić

**Affiliations:** 1 Division of Nephrology and Dialysis, Department of Medicine III, Medical University Vienna, Währinger Gürtel 18-20, A-1090 Vienna, Austria; 2 Institute of Biometrics, Center for Medical Statistics, Informatics, and Intelligent Systems, Medical University of Vienna, Vienna, Austria; 3 Department of Internal Medicine and Gastroenterology (IMuG), Hepatology, Endocrinology, Rheumatology, Nephrology and Emergency Medicine (ZAE), Klinikum Klagenfurt am Wörthersee, Klagenfurt, Austria

**Keywords:** Gitelman syndrome, Bartter syndrome, Transtubular potassium gradient, TTKG, Quality of life

## Abstract

**Background:**

Renal loss of potassium (K^+^) and magnesium (Mg^2+^) in salt losing tubulopathies (SLT) leads to significantly reduced Quality of Life (QoL) and higher risks of cardiac arrhythmia. The normalization of K^+^ is currently the most widely accepted treatment target, however in even excellently designed RCTs the increase of K^+^ was only mild and rarely normalized. These findings question the role of K^+^ as the ideal marker of potassium homeostasis in SLT. Aim of this hypothesis-generating study was to define surrogate endpoints for future treatment trials in SLT in terms of their usefulness to determine QoL and important clinical outcomes.

**Methods:**

Within this prospective cross-sectional study including 11 patients with SLTs we assessed the biochemical, clinical and cardiological parameters and their relationship with QoL (RAND SF-36). The primary hypothesis was that QoL would be more dependent of higher aldosterone concentration, assessed by the transtubular-potassium-gradient (TTKG). Correlations were evaluated using Pearson’s correlation coefficient.

**Results:**

Included patients were mainly female (82%, mean age 34 ± 12 years). Serum K^+^ and Mg^2+^ was 3.3 ± 0.6 mmol/l and 0.7 ± 0.1 mmol/l (mean ± SD). TTKG was 9.5/3.4–20.2 (median/range). While dimensions of mental health mostly correlated with serum Mg^2+^ (r = 0.68, *p* = 0.04) and K^+^ (r = 0.55, *p* = 0.08), better physical health was associated with lower aldosterone levels (r = -0.61, *p* = 0.06). TTKG was neither associated with aldosterone levels nor with QoL parameters. No relevant abnormalities were observed in neither 24 h-ECG nor echocardiography.

**Conclusions:**

Hyperaldosteronism, K^+^ and Mg^2+^ were the most important parameters of QoL. TTKG was no suitable marker for hyperaldosteronism or QoL. Future confirmatory studies in SLT should assess QoL as well as aldosterone, K^+^ and Mg^2+^.

## Background

Salt losing tubulopathies (SLT), most prominently Gitelman (GS) and Bartter-Syndrome (BS) represent the most frequent genetic tubular disorders [1]. BS includes different types of autosomal-recessive inherited disorders (Type I-V) resulting in severely reduced electrolyte reabsorption by the thick ascending limb of Henle while GS is caused by mutations in the thiazide sensitive sodium chloride co-transporter (NCCT) gene [2]. Typical clinical findings include renal loss of potassium (K^+^), magnesium (Mg^2+^) and calcium (Ca^2+^), leading to hypokalemia and metabolic alkalosis as well as increased renin and aldosterone levels.

Although earlier reports described patients with GS as mostly asymptomatic at presentation, Cruz et al. showed that in some patients quality of life (QoL) was markedly affected and comparable to other chronic diseases usually considered more severe such as diabetes mellitus or congestive heart failure [3–6]. Since SLTs originate from genetic mutations and causal therapies are not available yet, the therapy rather focuses on alleviation of symptoms via electrolyte supplementation. However, little is known about the relationship of biochemical parameters with QoL parameters.

The normalization of serum-potassium levels is currently the most widely accepted treatment target and therefore used as primary endpoint in clinical trials [7]. However, in even excellently designed randomized crossover trials, the increase of potassium with various treatment regimens was only mild and rarely within normal range [7]. Moreover, potassium correction may remain refractory due to underlying magnesium deficiency [8]. These findings question the role of serum K^+^ as the ideal marker of potassium homeostasis in SLT since simultaneous Mg^2+^ and Ca^2+^ wasting may also impact QoL. These electrolyte disbalances are further known to increase the risk for cardiac events in SLTs and in the general population [9]. Moreover, palpitations are a frequent symptom in SLTs which may per se affect QoL [6]. Therefore, additional thorough cardiac evaluation is essential not only to diagnose clinically inapparent cardiac abnormalities but also to exclude cardiac disease as a cause of reduced QoL.

The aim of this prospective cross-sectional hypothesis-generating study was to define surrogate endpoints for future treatment trials in SLT in terms of their usefulness to determine QoL and important clinical outcomes.

Our primary hypothesis was that higher aldosterone levels are associated with a poorer QoL and at the same time an increased risk for cardiac arrhythmias. As the measurement of aldosterone is costly, time-devouring and impracticable in daily routine, we additionally assessed the transtubular potassium gradient (TTKG) which was previously reported as a surrogate of aldosterone activity [10]. The TTKG is considered to quantify the renal potassium secretion by the cortical collecting duct, corrected for water reabsorption and was used as a parameter for guided diuretic treatment in other conditions with increased RAAS activity such as liver cirrhosis [11–13].

## Methods

### Study design

This is a prospectively designed cross-sectional hypothesis-generating study which was carried out in two different tertiary clinical centers in Austria (Medical University of Vienna and Klinikum Klagenfurt) after approval of the associated ethics committees (EK NR 1649/2017 and A 01/18). Medical files were screened for individuals with genetically or clinically diagnosed Bartter- or Gitelman-Syndrome. Patients entered the study after written informed consent assuring adherence to the declarations of Helsinki and Istanbul.

### Basic laboratory- and clinical findings

Basic demographic- and clinical findings were collected from electronic- and paper-based patient files. Intake of relevant medication (electrolyte supplements, diuretics) was recorded. Venous blood samples were collected for measurements of complete blood counts as well as blood chemistry including sodium, potassium, chloride, calcium, magnesium, albumin, creatinine, urea, uric acid, TSH and serum osmolarity. Urine electrolytes and urinary levels of urea, uric acid, creatinine, total protein, micro albumin and urine-osmolarity were measured from spot urine samples. TTKG was calculated according to the ratio of potassium in urine to serum adjusted for water reabsorption (ratio urine/plasma osmolarity).

### Study aim

The primary hypothesis was that higher plasma aldosterone concentrations and the additionally assessed TTKG were related to worse quality of life and higher risk for cardiac events and therefore better surrogate endpoints for treatment trials compared to serum potassium levels alone. Our secondary hypothesis was that other electrolyte disturbances frequently observed in SLTs, particularly magnesium and calcium levels were also related to QoL and cardiac abnormalities.

### Quality of life

RAND SF-36, a standardized questionnaire compound of 36 questions evaluating quality of life, was assessed in all patients [14]. The questionnaire consists of eight subdomains which are physical functioning, role limitations due to physical health, role limitations due to emotional problems, energy/fatigue, emotional well-being, social-functioning, pain and general health. We evaluated the RAND SF-36 questionnaire following the manufacturer instructions [14, 15]. We analyzed each quality of life subdomain separately. The score of each domain ranges from 0 to 100, with higher scores representing higher QoL. Physical quality of life and emotional quality of life were summarized to the physical (PCS) – and mental component scores (MCS). Summary scores were constructed by z-transformation and multiplication with corresponding coefficients of each domain. Coefficients for each domain are assumed to be country specific. The coefficients used in this evaluation are based on the general German population and were obtained from the cited source [16].

### Cardiological evaluation

Every patient was examined by transthoracic echocardiogram (TTE), and a 24-h electrocardiography. The following parameters were recorded: left atrial dimension, left ventricular dimension and mass, interventricular septum diameter, ejection fraction, longitudinal strain and the presence of wall movement disorder. The 24-h ECG was evaluated for basic rhythm, heart rate (minimum, maximum and mean), occurrence of arrhythmias and extrasystoles of ventricular or supraventricular origin. Episodes of tachycardia or bradycardia were noted when a heart rate of < 60 bpm or > 100 bpm occurred. Lowest/highest heart rate within 24 h as well as longest episode of bradycardia/tachycardia were documented. Before every 24-h ECG, a 12-lead resting ECG was performed. The corrected QT interval (QTc) was calculated according to the Bazett formula. A QTc of < 440 ms was considered normal [17, 18].

### Statistical analysis

Continuous variables were expressed as mean and standard deviation or median and absolute range, whichever was appropriate depending on the distribution of the data. Categorical variables were expressed as absolute and relative frequencies. Correlations were evaluated using Pearson’s correlation coefficient. In a second step, univariate and multivariate linear regression analysis was applied to determine laboratory parameters independently associated with aspects of QoL. Potential confounders of the effect size of the association between MCS/PCS and Mg^2+^/aldosterone were assessed using multivariate linear regression. A change in the effect size of > 10% was defined as significant. For multivariate analysis co-variables were selected on the basis of clinical relevance. According to recent literature a minimum of two subjects per variable are sufficient for interpretation of regression coefficients in multivariable linear regression [19]. Log normally distributed variables were log transformed. *P*-values < 0.05 were considered statistically significant. Statistical analysis was performed using commercially available software systems (Microsoft Office Excel; Microsoft Corp., Redmond, WA; SPSS; Version 25, SPSS Inc., Chicago, IL).

## Results

### Study participants

Eleven adult patients were screened for eligibility to enter the study, none of them refused participation. All patients were enrolled in the study between October 2017 and October 2018. Baseline characteristics of included patients are described in Table 1. Nine patients were diagnosed with GS, two with BS. Nine patients had their diagnosis genetically confirmed: seven patients with mutations in the SLC12A3 gene; two patients with mutations in the SLC12A1 gene. The majority of patients was female (*N* = 9 [81.8%]), mean age at study inclusion was 33.9 ± 12.4 years (mean ± SD). The median time since initial diagnosis was 10.0/2.0–26.0 years (median/range).

**Table 1:** Basic characteristics of the study population. All non-metric parameters are shown as absolute values and by percentages. Continuous variables are expressed as median and range. *: mean ± standard deviation (SD); ^+^median/range; K = potassium, Mg^2+^ = magnesium, Ca^2+^ = calcium, Na^+^ = sodium, TTKG = transtubular potassium gradient, N = number, PSD = potassium-sparing diuretic (eplerenone and spironolactone)

Basic characteristics	
Patients; N/%	11
Female; N/%	9 (81.8)
Age at diagnosis (years)*	24.1 ± 14.0
Age at study inclusion (years)*	33.9 ± 12.4
Time since diagnosis (years)^+^	10 (2–26)
Bartter-Syndrome; N/%	2 (22)
Gitelman-Syndrome; N/%	9 (78)
Medication; N/%	10 (91)
Patients with K^+^ and Mg^2+^ Supplements; N/%	7 (70)
PSD; N/%	6 (60)
Laboratory findings	
K^+^ mmol/L;*	3.3 ± 0.6
Ca^2+^ mmol/L;*	2.5 ± 0.2
Mg^2+^ mmol/L;* (n = 9)	0.7 ± 0.1
Na^+^ mmol/L;^+^	139/132.0–142.0
Osmolarity mosm/kg;^+^	286.0/282.0–291.0
Aldosterone pg/ml;^+^ (*n* = 10)	259.0.2/76.0–985.0
Urinary laboratory findings	
K^+^ mmol/L;^+^	33.0/9.0–120.0
Na^+^ mmol/L;^+^	62.0/17.0–142.0
Osmolarity mosm/kg;^+^	294.0/101.0–830
TTKG;^+^	9.5/3.4–20.2

### Routine treatment protocol

All patients were instructed to keep a diet rich in sodium, potassium and magnesium. Further, seven patients took K^+^ and Mg^2+^ supplements and potassium-sparing diuretics (PSD) were used in six patients (spironolactone [*N* = 4], amiloride [*N* = 1] eplerenone [N = 1], see Table 1). The median supplement based daily potassium- and magnesium intake was 64/0–304 mmol/d (median/range) and 7.5/0–30 mmol/d (median/range) respectively. Based on the clinic protocols, all patients were invited to ambulatory follow-ups every three months or upon clinical symptoms. The median number of ambulatory visits/blood collections at the center within the last year before study inclusion was 3/1–12 (median/range) respectively.

### Laboratory findings

Patients presented with mild hypokalemia (3.3 ± 0.6 mmol/L [mean ± SD]) and hypomagnesemia (0.7 ± 0.1 mmol/L). Serum osmolarity as well as sodium- and calcium levels were within the normal range (286.0/282.0–291.0 mosm/kg [median/range]; 139/132.0–142.0 mmol/L; 2.5 ± 0.2 mmol/L). The median aldosterone level was 259.0/76.0–985.0 pg/ml. Median urinary Na^+^ and K^+^ levels were 62.0/17.0–142.0 mmol/L (median/range) and 33.0/9.0–120.0 mmol/L (median/range) respectively. The median TTKG was 9.5/3.4–20.2 (median/range).

### TTKG is not associated with plasma aldosterone concentration

**Fig. 1:**
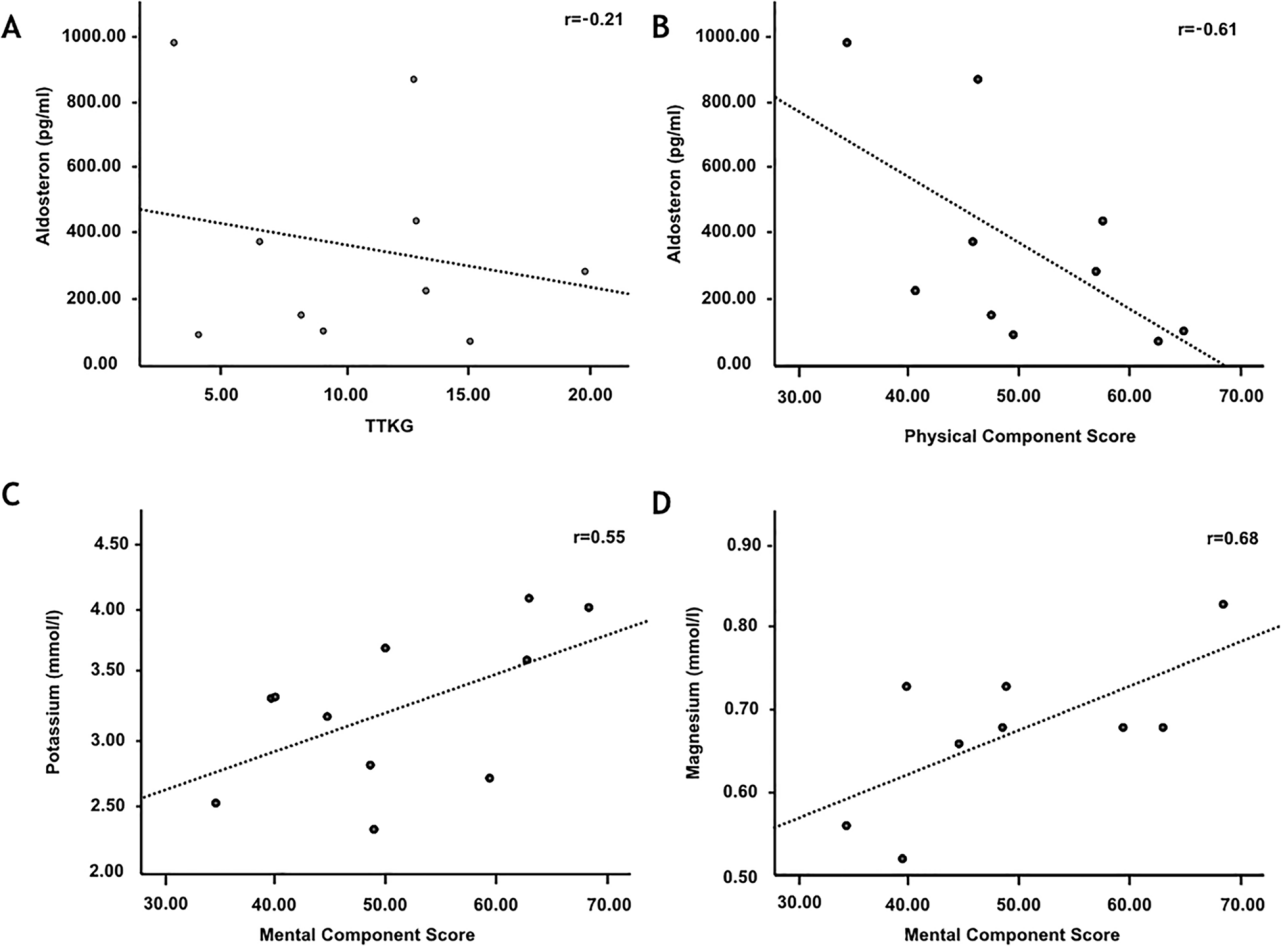
**a-d** Major correlations of biochemical findings and domains of QoL: **a**) The transtubular potassium gradient (TTKG) is not associated with plasma aldosterone levels. **b**) We found a trend towards higher plasma aldosterone levels and reduced Quality of Life domains of physical health, assessed by the physical component score. **c**) Higher serum potassium levels showed a trend towards increase of Quality of Life domains of mental health, assessed by the mental component score. **d**) Higher serum Mg^2+^ levels are significantly associated with an increase of Quality of Life domains of mental health, assessed by the mental component score

Of note, we could not document a correlation of serum potassium - (r = 0.43, *p* = 0.22) or TTKG (r = − 0.21, *p* = 0.56) with aldosterone (Fig. 1a). Likewise, using TTKG as a dichotomous variable according to the median of 10 (r = 0.06) as well as excluding cases with lower urine osmolality (*n* = 4), TTKG did not correlate with plasma aldosterone levels. TTKG was only associated with urinary potassium levels (r = 0.52). We further performed a sub analysis according to the use of potassium sparing diuretics (PSD). The correlation coefficient for TTKG with plasma aldosterone was − 0.12 (*p* = 0.86) in the PSD group compared to − 0.09 (*p* = 0.89) in the group without potassium sparing diuretics. Moreover, as shown in Table 2 we did not observe statistically significant differences in TTKGs, plasma- or urinary potassium levels and aldosterone levels in subjects with or without PSD.

**Table 2:** Plasma aldosterone, potassium and TTKG between patients with and without potassium sparing diuretics

	All	Patients with PSD	Patients without PSD	p
**Patients; N/%**	**11**	**6 (54.5)**	**5 (45.5)**	
Plasma K^+^ mmol/L;*	3.3 ± 0.6	3.5 ± 0.6	2.9 ± 0.9	0.126
Urinary K^+^ mmol/L;^+^	33/9–120	54/13–120	33/9–63	0.537
Aldosterone pg/ml;^+^ (n = 10)	259/76–985	442/96–985	156/76–379	0.222
TTKG;^+^	9.5/3.4–20.2	9.4/3.4–13.7	9.5/6.9–20.2	0.329

*: mean ± standard deviation (SD) ^+^: median/range; K = potassium, TTKG = transtubular potassium gradient, N = number, PSD = potassium-sparing diuretic (eplerenone and spironolactone)

### Quality of life

QoL scores of all eight domains are shown in Table 3. Higher scores in each subdomain represent better QoL parameters. Lowest scores were found in the categories role limitations due to physical health and fatigue (38.6 ± 42.4 [mean ± SD]; 40.9 ± 29.0 [mean ± SD]), whereas scores of social functioning and role limitations due to emotional problems showed the highest levels (70.5 ± 25.2, 60.6 ± 41.7). Both composite scores, the physical component score (PCS) as well as the mental component score (MCS) were rated similarly with mean scores of 50.0 ± 9.5 (mean ± SD) and 50.0 ± 10.7 (mean ± SD).

**Table 3:** a and b Correlation of biochemical parameters with the physical component score, mental component score and single domains of Quality of Life: Correlation are calculated using Pearson correlation. Significant findings are highlighted. K = potassium, Mg^2+^ = magnesium, Ca^2+^ = calcium, Na^+^ = sodium, TTKG = transtubular potassium gradient, N = number, PSD = potassium-sparing diuretic

	Domains of physical health									
	Physical Component Score		Physical Functioning		Role limitations- Physical		Pain		General health	
	R	p-value	R	p-value	R	p-value	R	p-value	R	p-value
TTKG > 10	0.28	0.39	0.40	0.22	−0.20	0.57	0.04	0.91	0.28	0.39
Plasma K^+^, mmol/l	− 0.28	0.41	− 0.22	0.52	−0.23	0.49	0.17	0.62	0.54	0.09
Plasma K^+^ > 3 mmol/l	− 0.33	0.33	− 0.20	0.57	−0.45	0.17	0.09	0.79	0.29	0.39
Urinary K^+^, mmol/l	−0.12	0.73	0.29	0.38	−0.36	0.28	− 0.19	0.56	−0.24	0.49
Urinary K^+^ > 20 mmol/l	0.11	0.74	0.43	0.18	−0.21	0.53	−0.04	0.91	0.11	0.74
Plasma Mg^2+^, mmol/l	0.31	0.42	−0.25	0.51	0.65	0.06	0.18	0.64	0.25	0.52
Plasma Ca^2+^, mmol/l	−0.30	0.39	−0.55	0.10	−0.42	0.23	0.03	0.93	0.28	0.44
Plasma Na^+^, mmol/l	0.28	0.41	0.61	0.05	0.26	0.44	0.04	0.90	−0.29	0.38
Urinary Na^+^, mmol/l	−0.35	0.32	−0.03	0.93	− 0.30	0.41	−0.35	0.32	− 0.52	0.13
Urinary Na^+^/K^+^ ratio	−0.14	0.70	−0.13	0.72	0.20	0.59	−0.25	0.49	−0.08	0.83
Aldosterone, pg/ml	−0.61	0.06	−0.50	0.14	**−0.74**	**0.02**	0.17	0.62	−0.54	0.09

	Domains of mental health									
	Mental Component score		Role limitations- Mental		Energy/Fatigue		Mental health		Social functioning	
	R	p-value	R	p-value	R	p-value	R	p-value	R	p-value
TTKG > 10	−0.41	0.21	−0.01	0.97	− 0.39	0.23	− 0.23	0.49	− 0.30	0.37
Plasma K^+^, mmol/l	0.55	0.08	−0.02	0.95	0.56	0.07	**0.70**	**0.01**	0.28	0.39
Plasma K^+^ > 3 mmol/l	0.22	0.52	−0.27	0.42	0.33	0.32	0.45	0.17	−0.05	0.89
Urinary K^+^, mmol/l	−0.47	0.14	−0.08	0.81	−0.48	0.14	−0.28	0.39	−0.42	0.19
Urinary K^+^ > 20 mmol/l	**−0.66**	**0.03**	0.04	0.89	−0.83	0.002	−0.56	0.07	−0,34	0.31
Plasma Mg^2+^, mmol/l	**0.68**	**0.04**	0.48	0.19	0.40	0.28	0.48	0.28	**0.74**	**0.02**
Plasma Ca^2+^, mmol/l	−0.06	0.87	−0.47	0.17	0.11	0.76	−0.02	0.95	−0.11	0.75
Plasma Na^+^, mmol/l	−0.06	0.85	0.43	0.19	−0.31	0.35	−0.05	0.89	0.09	0.78
Urinary Na^+^, mmol/l	−0.37	0.29	0.09	0.81	−0.55	0.10	−0.39	0.26	−0.13	0.73
Urinary Na^+^/K^+^ ratio	0.22	0.53	0.33	0.36	0.06	0.89	0.17	0.65	0.25	0.49
Aldosterone, pg/ml	−0.26	0.46	−0.49	0.15	−0.05	0.89	−0.14	0.71	−0.45	0.19

### Single domains of QoL and laboratory findings

Table 3 a and b illustrate the association of biochemical parameters with single and composite domains of QoL. Higher serum K^+^ significantly correlated with emotional well-being (r = 0.70, *p* = 0.01) and showed a trend for better general health (r = 0.54, *p* = 0.09) and less fatigue (r = 0.56, *p* = 0.07). Aldosterone levels and limitations due to physical health (RP) were negatively correlated (r = − 0.74, *p* = 0.02). Serum Mg^2+^ levels had a significant positive correlation with social functioning (r = 0.74, p = 0.02). Further, Mg^2+^ showed a trend towards less limitations due to physical health (r = 0.65, *p* = 0.06). In contrast, neither the TTKG alone nor the TTKG after exclusion of cases with low urine osmolality were significantly correlated with QoL domains. Serum calcium was not related to single domains of QoL. We did not find a significant correlation between QoL domains and the cumulative daily doses of the magnesium- (PCS: r = − 0.10, *p* = 0.77; MCS: r = − 0.16, *p* = 0.66) and potassium supplementations (PCS: r = − 0.10, *p* = 0.78; MCS: r = − 0.29, *p* = 0.43). The same applied for the number of clinic visits/blood samples within the last year (PCS: r = − 0.35, *p* = 0.29; MCS: r = − 0.49, *p* = 0.12).

### Component scores of QoL and laboratory findings

To assess QoL in a more general way we calculated the physical and mental component scores according to standard methodology. As shown in Fig. 1 and Table 3, dimensions of mental health were significantly correlated with Mg^2+^ (r = 0.68, *p* = 0.04) and showed a trend towards higher serum K^+^ levels (r = 0.55, *p* = 0.08). Further, we found a trend towards better physical health with lower aldosterone levels (r = − 0.61, *p* = 0.06). Applying univariate linear regression analysis, the regression coefficient for magnesium and MCS was statistically significant (1.11, [95% CI: 0.05–2.17]; *p* = 0.042). Inclusion of TTKG or serum potassium in a multivariate model did not significantly change the regression coefficients. We observed a clinically relevant trend between PCS and aldosterone (− 0.14, [95%CI, − 0.28-0.06], *p* = 0.059). Inclusion of serum potassium or TTKG did not significantly change regression coefficients. However, inclusion of aldosterone into the MV model significantly increased the regression coefficient for magnesium and MCS (1.88, *p* = 0.01). These findings support the hypothesis that both magnesium and aldosterone may serve as valuable and synergistic markers of QoL parameters.

### Cardiological evaluation

Cardiological work-up consisted of an echocardiogram and 24-h electrocardiography. The study population showed normal mean left atrial and ventricular dimensions of 33.1 ± 3.6 mm and 44.7 ± 4.3 mm without signs of hypertrophy (interventricular septum was 8.0/7.0–1.1 mm [median/range]). Right ventricular dimensions were within normal ranges in all patients. The left ventricular ejection fraction was always above 55% and the longitudinal strain was normal. We did not find any episodes of arrhythmia with 24-h-electrocardiography. All patients had sinus rhythm with a mean heart rate of 87 bpm (range 53–125). Three patients had sinus tachycardia for more than ten minutes. The median QTc was 425/416-498 ms (median/range) respectively. Two patients had a prolonged QTc (470 ms and 454 ms) and in four patients a U-wave was found.

## Discussion

In comparison to previous reports, this is the first study showing significant correlations of domains of QoL with laboratory findings in SLTs [3, 20]. Higher plasma aldosterone levels correlated significantly with higher limitations due to physical health and showed a trend towards an overall worse physical health perception. Whereas better mental health was significantly correlated with higher serum Mg^2+^ we also found a trend towards higher serum K^+^ levels. Our findings emphasize that serum potassium alone does not correlate with the overall individual disease burden assessed by QoL instruments and is therefore an insufficient treatment endpoint in randomized controlled trials in SLTs.

Frequent ambulance visits and blood tests, as well as high doses of supplements and the related side effects may be considered a factor contributing to the reduced QoL in SLT patients. However, in our study neither the number of ambulatory tests nor the dose of supplement consumed was correlated with QoL.

The association of Mg^2+^ with mental health is in line with previous literature reporting efficacy of Mg^2+^ supplements in relieving anxiety and stress and its use as an analgesic sparing substance in anesthesiology [21, 22]. Moreover, the use of slow-releasing magnesium formulation medications led to an increase of patient reported outcomes in a cohort of GS patients [23].

Since its first description in rat micro puncture studies, TTKG has been considered a reliable surrogate of aldosterone activity [10]. In our study, TTKG did not prove a useful parameter of QoL. Moreover TTKG was not associated with plasma aldosterone concentrations, even after exclusion of patients treated with potassium sparing diuretics in a subgroup-analysis. This is in line with a prior study assessing TTKG in drug-induced hyperkalemia which found that TTKG values did not differ between patients with potassium sparing drugs and patients with hyperkalemia of other causes [24]. More recent publications have also questioned the ability of TTKG to reflect aldosterone activity. For instance, Kawada et al. did not detect an association of TTKG with aldosterone in hypertensive patients [25]. Kamel et al. provided a possible explanation for these findings as urea recycling in the medullary collecting duct leads also to a significant amount of potassium wasting and TTKG may therefore rather reflect combined aldosterone and vasopressin activity [26]. The authors, who were also part of the team first describing the TTKG assume that the micropuncture related interruption of urea recycling may explain the discrepancies between the in vivo data and emerging clinical findings [26]. However, a partial effect or a combination of concomitant volume depletion leading to decreased sodium delivery to the collecting duct and an impaired function of the ENaC may contribute to discrepant results regarding TTKG and aldosterone in some of the studies. From a pathophysiologic perspective, decreased sodium delivery to the ENaC is an unlikely confounder in salt-losing tubulopathies. Also, in our study we observed only two patients with a urinary sodium level < 25 mmol/l, both without concomitant PSD. We can therefore exclude a relevant effect of decreased sodium delivery to the ENaC in the overall cohort, which may have partially explained the lack of an association between TTKG and aldosterone in some of the before mentioned studies.

Previous case reports described a high risk for cardiac events including aborted sudden cardiac death events in SLT [9]. Suggested potential mechanisms besides hypokalemia include exercise induced left ventricular dysfunction leading to reduced cardiac index as well as paradoxical QTc prolongation and subsequent cardiac dysfunction during nocturnal vagal stimulation [9]. In our study non-invasive cardiological diagnostics showed prolonged QTc intervals in two patients and U waves in four patients but no evidence for cardiac ventricular dysfunction or prolonged arrhythmia. Therefore, we can safely exclude any impact of clinically inapparent cardiac abnormalities on QoL. However, it should be noted that these patients had only moderate hypokalemia and hypomagnesemia and that our study protocol did not include stress-induced cardiac evaluation. Our results may therefore not be comparable to the beforementioned study.

Some limitations must be considered when interpreting our study. First, there are inherent limitations related to studies in orphan diseases. Due to the low prevalence of SLTs, prospective investigations are difficult to perform. This trial was therefore designed as a hypothesis-generating study needing testing in a confirmatory study and in this comparably small cohort, significant associations of laboratory parameters and QoL domains were found. We have also corrected our findings with multivariate linear regression models, which provide stable regression coefficients even in smaller sample sizes [19]. However, it must be pointed out, that while the obtained results may provide a valuable basis for future trials they cannot be interpreted as confirmatory and a validation in a larger data set is necessary. Second, the cross-sectional study design does not allow a causative interpretation of our findings. Third, our study cohort consists of both BS and GS patients, which may seem heterogeneous from a pathophysiological perspective. The aim of the current study is not the underlying pathophysiology but the clinical phenotype and its QoL related features with distinct focus on the omnipresent role of hyperaldosteronism in SLTs. Moreover, there is broad evidence and consensus that despite the differences expected to be present in tubular electrolyte handling in those syndromes, clinical phenotypes are often undistinguishable with up to 30% of BS patients having a GS phenotype [1, 27–29]. Further, various conditions present in SLTs can lead to an increase and/or decrease of plasma aldosterone and plasma aldosterone is therefore not a specific marker of potassium depletion [30]. While the current study is not designed to define treatment thresholds for plasma aldosterone concentration it provides evidence that a composite laboratory endpoint of potassium, magnesium and aldosterone concentration may be reflecting QoL more accurately than serum potassium alone.

## Conclusions

To conclude, this is the first study reporting significant associations of biochemical parameters and QoL in patients with SLTs and provides a valuable basis for novel treatment goals and future trials. Confirming our primary hypothesis, mental health was mostly associated with higher Mg^2+^ levels and trends towards better physical aspects of QoL in patients with lower plasma aldosterone were found.

Future treatment trials in SLT should therefore aim at increasing domains of QoL by influencing serum levels of magnesium, potassium and aldosterone.

## Abbreviations

BPM: Beats per minute
BS: Bartter-Syndrome
Ca^2+^: Calcium
E: Energy/Fatigue
GH: General health
GS: Gitelman-Syndrome
K^+^: Potassium
MCS: Mental component score
Mg^2+^: Magnesium
Ms.: Milliseconds
Na^+^: Sodium
PCS: Physical component score
PF: Physical functioning
PSD: Potassium-sparing diuretic
QoL: Quality of Life
RAAS: Renin-Angiotensin-Aldosterone System
RP: Role limitations due to physical problems
RE: Role limitations due to emotional problems
SF: Social functioning
TTKG: Transtubular potassium gradient
